# The Spanish language version of the TAPS tool: protocol for a validation and implementation study in primary care

**DOI:** 10.1186/s13722-023-00423-9

**Published:** 2023-11-16

**Authors:** Jan Gryczynski, Katherine Sanchez, Steven B. Carswell, Robert P. Schwartz

**Affiliations:** 1 Friends Research Institute, COG Analytics, Baltimore, MD USA; 2 Baylor Scott and White Research Institute, Dallas, TX USA

**Keywords:** SUD screening, Spanish, Validity, Linguistically accurate, Culturally relevant, Primary care

## Abstract

**Background:**

The TAPS Tool (“Tobacco, Alcohol, Prescription drug, and illicit Substance use”) is a screening and brief assessment for detecting unhealthy substance use in healthcare settings that was developed by the National Institute on Drug Abuse Clinical Trials Network and validated in a multisite study. Our team developed a Spanish language version of the TAPS Tool that supports provider- and self-administration screening using a mobile/web-based platform, the TAPS Electronic Spanish Platform (TAPS-ESP).

**Methods:**

This article describes the protocol and rationale for a study to validate the TAPS-ESP in a sample of Spanish-speaking primary care patients recruited from a network of community-based clinics in Texas (target *N* = 1,000). The TAPS-ESP will be validated against established substance use disorder diagnostic measures, alternative screening tools, and substance use biomarkers. The study will subsequently examine barriers and facilitators to screening with the TAPS-ESP from a provider workflow perspective using qualitative interviews with providers.

**Discussion:**

Validating a Spanish language version of the TAPS Tool could expand access to evidence-based, linguistically accurate, and culturally relevant substance use screening and brief assessment for an underserved health disparity population.

*Trial registration***:** The study was registered with www.clinicaltrials.gov: NCT05476588, 07/22/2022.

## Background

Substance Use Disorders (SUDs) for tobacco, alcohol, illicit drugs, and non-medical use of prescription drugs contribute to substantial public health and social problems, and minority populations bear a great share of the disease burden [1–7]. Substance use can have major consequences for health, including overdose death, intoxication-related injury, and soft tissue infections from drug injection. SUDs can exert considerable public health burden in morbidity and mortality related to their long-term sequelae (e.g., cirrhosis from alcohol, lung and esophageal cancers from tobacco, hepatitis C infection from injection drug use). However, significant health consequences of unhealthy substance use can manifest even with less severe and sub-diagnostic SUDs.

For these reasons, the US Preventive Services Task Force now recommends that all adults receive routine screening for all substances as part of primary care. While screening for tobacco and alcohol have long been recommended on the basis of rigorous evidence synthesis from randomized trials [8–10], the Task Force recently updated its position to recommend screening for illicit drugs and non-medical prescription drug misuse [11]. This change was prompted by a growing body of research demonstrating the ability of brief screening tools to validly detect unhealthy substance use in large, methodologically rigorous studies. The US Surgeon General has strongly endorsed implementing substance use screening and intervention in health care systems [12].

Even with the well-described comorbidities of SUD with serious medical and mental health conditions [13], many primary care settings fail to systematically screen for unhealthy substance use, or do not use best evidence screening tools [14]. However, recent research has yielded more empirically-validated screening tools to detect substance use problems in primary care [15–18]. Although substance use problems are prevalent in primary care populations, most individuals who access primary care do not seek treatment in the behavioral health service system on their own [19].

Limited English Proficiency is a barrier to quality behavioral health care. Demographic changes in populations served by the large nationwide network of Federally Qualified Health Centers (FQHCs) show a rise in substance use by young, low-income, racial and ethnic minorities, especially Hispanic Americans [20]. Individuals with limited English proficiency are less likely to self-identify a need for behavioral health services, resulting in a longer duration of untreated disorders [21]. Accurate screening, diagnosis, and treatment are entirely dependent on a linguistically-accurate and culturally-appropriate identification and assessment, especially for sensitive topics such as substance use. Language accessibility is essential to the treatment of mental and physical health disorders [22], the lack of which is a significant contributor to health disparities, lack of patient satisfaction in healthcare, and poor quality patient education and understanding of their disorder [23, 24]. Health disparities among racial minorities are amplified in the presence of comorbid substance use and psychiatric disorders [25, 26]. Despite the clear need, few substance use screening tools have been validated with Spanish-speaking populations.

There are over 60 million Hispanics in the US, comprising 18.5% of the population, making it the largest and fastest growing minority group [27]. Three-in-four (73%) Hispanics speak Spanish at home, and nearly 40% are Spanish dominant (or Spanish preferred), making it the most spoken non-English language in the US [28]. Limited English proficiency, limited health literacy, geographic inaccessibility, and lack of medical insurance are all more common among immigrant minority groups of low socio-economic status [29].

National epidemiological survey data shows that, among Hispanics in the US, rates of past year substance use were 19.8% for tobacco, 57.9% for alcohol use, 13.5% for cannabis, and 8.2% for illicit drug use for non-medical use of prescription drugs [30]. While these rates are similar to those of the general population, Hispanics are significantly less likely than non-Hispanics to access substance use treatment when they need it and are less likely to perceive a need for it despite meeting standard clinical thresholds for substance use problems [31]. Thus, Hispanics experience disparities in the identification of SUDs and in access to care. Hispanics experience a disproportionate burden of disability associated with mental health disorders because of these disparities [32, 33].

The past decade has increased emphasis on the role of technologies in patient assessment and care, with health systems adopting electronic health records (EHRs) and other health information technologies [34]. More recently, the COVID-19 pandemic has led to a rapid adoption of health communication technologies, with many primary care providers quickly building upon their telehealth infrastructure to deliver more services remotely. Valid screening and diagnostic technologies that can be integrated into EHR systems have great potential for improving patient care and reducing health disparities, both for services provided in-clinic and remotely.

Although a number of substance use screening tools are available, many have shortcomings that may make them unsuitable for the modern primary care environment. Some long-established screeners like the CAGE for alcohol or the Fagerstrom Test for Nicotine Dependence focus narrowly on a single substance. Other tools (e.g., the Drug Abuse Screening Test, or DAST) do not differentiate between types of substances. Others like the World Health Organization’s ASSIST have been criticized as being too long. The need for a single, brief instrument without these shortcomings drove the National Institute on Drug Abuse Clinical Trials Network (NIDA-CTN) to develop the “Tobacco, Alcohol, Prescription medication, and illicit Substance use screening Tool” (or TAPS Tool) [16, 18, 35, 36]. The TAPS Tool is a two-stage screening and brief assessment instrument based on the WHO ASSIST-Lite [37] that first screens the four broad substance use categories (tobacco, alcohol, prescription drug misuse, and illicit substances), then branches to the brief assessment in which the patient is assessed for more specific risks related to an expanded array of substances (tobacco, alcohol, cannabis, cocaine, methamphetamines, prescription stimulants, heroin, prescription opioids, sedatives, and other substances). The 4-item screener triggers brief assessment on these substance categories, and each substance yields a score of 0–3 (except alcohol, which is scored on a 0–4 range), which conveys to providers whether an intervention is indicated (a score of 1 suggests sub-diagnostic problem use; a score of 2 suggests substance use disorder). The TAPS Tool was validated against established diagnostic assessments in interviewer- and self-administered formats in a large, multi-site study [16]. However, the original TAPS study was only conducted using an English language version. Only 11.7% of the sample reported Hispanic ethnicity, and the ability to speak and read English was an inclusion criterion.

There has been surprisingly little existing research within the US that has validated substance use screening in Spanish. Saitz et al. examined Spanish-language versions of the CAGE and AUDIT alcohol screeners in a primary care practice among 210 Hispanic patients [38]. Over 1 in 3 participants met criteria for alcohol use disorder, defined at the time as alcohol abuse or dependence under the Diagnostic and Statistical Manual of Mental Disorders, 4^th^ edition (DSM-IV). The widely used AUDIT proved unsuitable for this sample, with a detection sensitivity of only 51% for alcohol use disorder, while the CAGE showed good sensitivity and specificity for these diagnoses (80% and 93% at a cut-point of 2, respectively).

Bedregal et al. studied test–retest reliability of Spanish-language translations of the Drug Abuse Screening Test-10 (DAST-10) and Reduced/Annoyed/Guilty/Start (RAGS) screeners [39]. Of 222 Hispanic participants recruited, 78 completed both interviews. The measures were administrated in counterbalanced order. Both screens were found to have unidimensionality and were able to differentiate between participants with drug vs. alcohol vs. no substance use problems [39]. McCabe et al. examined the factor structure of a Spanish-language version of the College Alcohol Problems Scale in a sample of 125 Spanish-speaking undergraduates [40]. The researchers found acceptable fit for a two-factor model and acceptable internal consistency (α = 0.76 and 0.73 for the personal and social problems subscales, respectively). However, this scale is considered to be more of a research measure for problematic alcohol use in college students, and not suitable for general screening in primary care [40].

In a recent study conducted in the US and Spain, researchers used item response theory to construct an English and Spanish version of a computerized adaptive substance use scale (the CAT-SUD), with Spanish-speaking participants drawn from both countries [41]. The calibration and validation study found 11 items that had high correlation with the full 168-item scale (r = 0.91) and able to identify SUDs (AUC = 0.85). Like most other validation studies, this study used the Spanish-language translation of the Composite International Diagnostic Interview (CIDI) SUD items as the diagnostic standard [41]. The prior research on Spanish-language validation of substance use screening tools in the US has shown promising reliability and validity. However, this literature is characterized by small samples, has focused on a limited number of substances, and has mostly been fielded in non-primary care populations. The current study builds on this prior literature with a validation study.

Our team previously developed a Spanish-language version of the TAPS Tool and designed a technology platform to deliver the screening and brief assessment to a Spanish-speaking, health disparity population in community health centers. We refer to the resulting package as the TAPS-Electronic Spanish Platform (TAPS-ESP), which includes resources for both traditional interviewer-administered and electronic self-administered formats. Findings from our small feasibility, acceptability, and preliminary validation study of TAPS-ESP support the utility of the TAPS Tool, which yielded similar rates of detection of substance use as Spanish-language versions of well-established, widely used measures. Moreover, participants reported high acceptability of the TAPS Tool, which is consistent with the findings of the English language multisite validation study [42]. Since Hispanics with SUDs significantly underutilize specialty treatment due to barriers stemming from stigma, lack of social support, cultural factors, and family conflict [43–45], the next stage for this type of research is to conduct a full validation of the TAPS-ESP in a large sample to build its evidence base for Spanish-speaking primary care patients.

## Methods

### Overview

This study will validate the TAPS-ESP in both interviewer- and self-administered formats, largely mirroring the methodology of the seminal English language validation study.

This project is motivated and informed by the principles of the Conceptual Framework for Advancing Health Disparities Research within the Health Care System described by Kilbourne et al. [46]. Briefly, this framework posits a nested structure of factors that perpetuate health disparities within the health services arena, including healthcare system factors (organization, financing, culture, etc.), within which patient and provider factors intersect at the clinical encounter. It is at this level that patient-provider communication and cultural competence are paramount. The availability of linguistically-accurate screening and brief assessment resources (especially if paired with clinical decision support) has the potential to improve provider competency in addressing substance use problems, while elevating patients’ receptivity to provider communication about behavior change, harm reduction, or referral. The framework further organizes disparities-focused services research into phases of (a) detection (identifying, measuring, and tracking disease in a defined population), (b) understanding (determinants and perpetuators of disparate service access and outcomes, including at the level of patients, the clinical encounter, and the health care system), and (c) reduction (translation and dissemination of evidence-based practices and policies). The TAPS-ESP project is focused on the detection phase but will also target the understanding phase by considering acceptability of the TAPS-ESP among clinic patients and perceived barriers and facilitators to its use by providers. In a broad sense, the TAPS-ESP effort seeks to directly address gaps in access to evidence-based substance use screening for a health disparity population by removing linguistic barriers and developing the evidence base with the target population.

### Setting and recruitment

The TAPS-ESP validation study will be conducted at Baylor Scott and White Health (BSWH), the largest non-profit health system in Texas and one of the largest in the US. The BSWH system includes multiple primary care sites across the Dallas/Ft. Worth metroplex. Participants will be recruited from up to seven primary care sites of the Community Clinic network operated by BSWH. Depending on the clinic site, Hispanic patients make up 51%-80% of the patient population, and the majority are Spanish-dominant or Spanish-preferred. All research staff will be bilingual, native Spanish speakers. The providers in the clinic will deliver care in Spanish, some as native Spanish speakers, some with interpreters present.

We will use a multi-pronged recruitment strategy, including (a) direct invitations by research staff in the clinic waiting room, (b) direct invitations from telehealth visit schedules, (c) direct provider referrals, and (d) patient-initiated contact from advertisements posted in the clinic sites. Within these approaches, we will focus recruitment on the general primary care population, as well as targeting patients who are engaged with the behavioral health services provided within BSWH (where prevalence of substance use is likely to be higher).

*Inclusion criteria* will be: (1) age 18 or older; (2) BSWH patient; and (3) Spanish-language preferred (with the ability to read Spanish).

*Exclusion criteria* will be: (1) unable to provide informed consent (e.g., due to impairment or psychosis). All research activities (e.g., informed consent discussion and interview) will occur in Spanish. Participants will sign an IRB-approved Spanish language consent form.

### Study procedures and informed consent

**Fig. 1:**
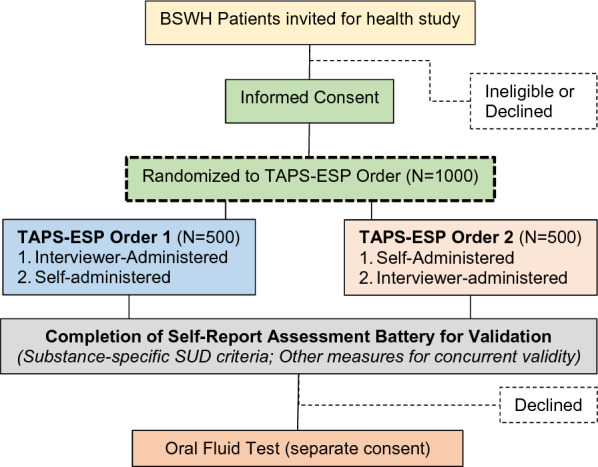
Recruitment and procedures flow for the validation study

Procedures for the validation study involve a one-time research visit. All participants will complete the TAPS-ESP in both interviewer- and self-administered formats. However, to test for administration order effects, the order of the format will be determined at random, see Fig. 1 for SPIRIT flow diagram. Randomization addresses confounding by administration order, thereby allowing an unbiased comparison of self- and interviewer-administration formats (both of which are widely used in clinical care). This design was used in the original TAPS study and our prior work [42]. Randomization is automated, with minimal extra time burden.

After completing the TAPS-ESP in both formats, participants will complete a battery of self-report measures for validation purposes, as well as a brief satisfaction and acceptability questionnaire. Self-report measures will be interviewer administered only. Participants will receive $40 for completing the self-report portion of the validation study, which is expected to take about 1.5–2.0 h for participants who report polysubstance use. Because this is a validation study, participants will be informed at the outset that each measure must be asked in its entirety, and to expect some redundancy in the questions. Nevertheless, the assessment may be slightly shorter in duration for participants with limited substance use due to skip patterns that exist within each measure.

At the conclusion of the self-report measures, participants will be invited to provide an oral fluid cheek swab, which will undergo rapid assay testing for biomarkers of recent substance use. Participants will be asked to sign a separate consent for the oral fluid testing and will receive an extra $10 for providing a sample. This approach will be undertaken to ensure that self-report measures are assessed free of bias that may occur if participants know ahead of time that they will be tested. This will serve as a test of the veracity of the self-report information obtained from participants. We will track refusal to consent to the oral fluid test component and will analyze these data under different assumptions (e.g., completers only; refusals imputed as positive). Participants will also be asked about medications they are taking as prescribed in order to account for medicines that may be reactive with the tests.

### Measures

All measures will be administered in Spanish-language translations. These measures were selected because they represent widely used comparative standards for which Spanish translations are available. Thus, for this reason and for consistency with the broader screening literature, our primary analysis will focus on validating the TAPS-ESP against the DSM-5 SUD diagnostic criteria. Other screening tools will be examined with respect to establishing concurrent validity. The measures are summarized in Table 1, and each is described below.

**Table 1:** Summary of primary and secondary substance use measures for TAPS-ESP validation

Measure	Alcohol	Tobacco	Cannabis	Illicit Opioids	Rx Opioid	Rx Stim-ulants	Metham-phetam	Cocaine	Hallucin-ogens	Sedatives
TAPS-ESP	✔	✔	✔	✔	✔	✔	✔	✔	✔	✔
Primary Criterion Standard (DSM-5 Diagnostic Criteria)										
CIDI items mapped to DSM-5	✔	✔	✔	✔	✔	✔	✔	✔	✔	✔
Secondary Self-Report Measures (concurrent validity)										
ASSIST	✔	✔	✔	as single category		as single category		✔	✔	✔
DAST-10			Problems covering all drugs other than tobacco/alcohol; does not differentiate specific drugs							
CAGE	✔									
AUDIT	✔									
FNDS		✔								
Biomarker										
Oral Fluid/Saliva test	✔	✔	✔	cross-reactive		cross-reactive		✔		most

✔ included in screening measure

#### Primary criterion standard for validation

*Modified World Mental Health Composite International Diagnostic Interview (CIDI):* The CIDI is a comprehensive instrument used to assess mental health disorders based on criteria in the International Classification of Diseases (ICD-10) and the Diagnostic and Statistical Manual of Mental Disorders (DSM-IV and DSM-5). Substances covered in the CIDI are alcohol, tobacco, and nine substance categories. As is the methodological standard in the field and as done in our prior research [42], we will administer a modified version of the CIDI consisting of the subset of items mapping to DSM-5 substance use disorder (SUD) criteria, asked for each endorsed substance class. We will examine categories of unhealthy use (1 DSM criterion) and SUD (2 + criteria), using the CIDI as the “gold standard” for identifying SUDs (i.e., patients at higher risk) [47, 48]. This is consistent with the methodology of the original English-language TAPS study and many other validation studies. The CIDI items mapped to the DSM-5 criteria for each substance will serve as the primary criterion standard.

#### Secondary self-report measures

Several secondary measures will be administered for the purposes of assessing the concurrent and convergent validity of the TAPS Tool. Many of these tools are limited to one substance (e.g., CAGE, FTDN), or assess for problems that are not substance-specific (e.g., DAST-10).

*Alcohol, Smoking, and Substance Involvement Screening Test (ASSIST):* The ASSIST was developed for the World Health Organization to screen for alcohol, tobacco, and drug use in medical care settings. For each substance, the use dimensions include lifetime use, past 3-month use, urges or cravings to use, and adverse consequences from use, as well as concerns expressed by family or friends about use (lifetime, past 3 months), failed attempts to control, cut down, or stop use (lifetime, past 3 months), and drug injection (lifetime, past 3 months). The ASSIST provides substance-specific risk scores, 9 substance classes, with scores of 1–3 corresponding to *low risk*, 4–26 (10–26 for alcohol) to *moderate risk*, and 27 or higher to *high risk* [49]. As a WHO instrument, the ASSIST is available in many languages, including Spanish. A number of psychometric or validation studies of the Spanish language version of the ASSIST have been conducted [50].

*Drug Abuse Screening Test (DAST-10):* The DAST-10 is a ten-item yes/no screen for general drug use problems (not including alcohol). Each item is worth one point, and respondents are tiered into risk categories based on score, with more intensive assessment recommended at a score of 6 or higher. It has been widely used in the substance use field but does not distinguish between types of drugs used. A Spanish language version has been developed [39].

*CAGE:* The CAGE is a rapid alcoholism screening test comprised of four yes/no questions (Cut Down, Annoyed, Guilty, Eye-Opener) [51]. A Spanish language version was validated in both Spain and the US, where it performed well in identifying DSM-IV alcohol abuse or dependence among Latino/a primary care patients [38]. An affirmative response to any of the items was sensitive in identifying DSM-IV alcohol abuse or dependence.

*Alcohol Use Disorders Identification Test (AUDIT):* The AUDIT is a 10-item questionnaire which covers the domains of alcohol consumption, drinking behavior, and alcohol-related problems. It was developed from a six-country World Health Organization collaborative project as a screening instrument for hazardous and harmful alcohol consumption [52]. Responses to each of the 10 questions are scored according to a frequency rating of 0 (never) to 4 (daily), giving the entire questionnaire a possible score of 40. A score of 8 or more indicates harmful or hazardous alcohol use. A Spanish language version of the AUDIT was tested within the US in a small study, where only 51% of patients with alcohol use disorder were identified at the standard cut-point [38]. Thus, this widely used tool may not be optimal for Spanish-speaking populations in the US. We will administer the AUDIT to directly quantify the differences with the TAPS Tool in identifying alcohol use disorder.

*Fagerstrom Test for Nicotine Dependence (FTND):* The FTND is comprised of six questions scored on a point system, with total scoring summing between 0 and 10. Higher scores indicate heavier reliance on nicotine. The FTDN has been widely used in the tobacco field for decades [53]. It has been found to be internally consistent and an acceptable way to measure nicotine/tobacco dependency. The FTND has been translated into Spanish and validated. [54]

#### Biomarkers of substance use

*Oral Fluid Tests* As done in the original TAPS study [16], oral fluid assay testing will be used to detect recent use of substances including alcohol, nicotine, cannabis, cocaine, amphetamines, sedatives (benzodiazepines), and opioids (heroin/morphine metabolite, oxycodone, buprenorphine, methadone, and fentanyl). Participants will be asked about prescribed medications to account for cross-reactive metabolites. Although oral fluid only captures recent use (within the past few days for most substances), it is useful as an objective biomarker to validate self-report. The study will use the Orawell® 12-panel saliva test plus alcohol and the NicDetect oral fluid test for cotinine. According to the product inserts, detection cut-offs are as follows: amphetamines (25 ng/mL), methamphetamine (25 ng/ML), barbiturates (25 ng/mL), benzodiazepines (10 ng/ml), cocaine (20 ng/mL), morphine (10 ng/mL), THC (20 ng/mL), oxycodone (40 ng/mL), methadone (30 ng/mL), buprenorphine (10 ng/mL), fentanyl (10 ng/mL), tramadol (25 ng/mL), alcohol (0.02% BAC), and cotinine (30 ng/mL).

#### Process Measure

*Satisfaction and Acceptability Questionnaire* After completing the TAPS-ESP (and prior to completing the other measures), participants will be asked to complete a brief questionnaire to gauge feasibility and acceptability of the TAPS-ESP. This survey will mirror the feasibility/acceptability questions used in our prior work. Sample questions include “How much do you agree with the following statements?” (rated on a 5-point Likert-type scale; e.g., “not at all true” to “very true”): I would be willing to answer questions like these at my doctor’s office every year; I answered the questions as honestly as I could; I think my friends and family would answer these questions honestly at their doctor’s office; The questions were easy to understand; The touchscreen tablet was easy to use.” In addition, participants will be asked about their preferences and comfort with different ways in which TAPS-ESP information could be used clinically (e.g., automatic provider notification regarding patients’ screening scores), and preferences for intervention among participants who screen positive (e.g., provider-delivered vs. interactive computerized intervention).

### Statistical analysis

Validation of the TAPS-ESP will follow the roadmap provided by the original English language TAPS study [16]. First, validation of the TAPS-ESP will be done for the broad substance categories in the TAPS-1 screening portion of the Tool (tobacco, alcohol, illicit drugs, non-medical use of prescription drugs). Second, we will conduct validation with the entire TAPS Tool for more specific substance classes (tobacco, alcohol, cannabis, cocaine, illicit amphetamines/methamphetamines, non-medical use of amphetamine-type stimulant medications, heroin/illicit opioids, non-medical use of opioid analgesics, and non-medical use of sedative medications). In addition, we will conduct analyses that combine logical categories (for example, any SUD other than tobacco; opioids inclusive of heroin/illicit opioids and non-medical use of opioid medications; all illicit drugs other than cannabis).

#### Analyses of TAPS-ESP performance

The TAPS-ESP scores will be validated against the CIDI as the primary diagnostic reference standard. Secondary screeners and assessments will be similarly examined from the standpoint of concurrent validity. Statistical analysis of TAPS-ESP screening test performance will employ a receiver operating characteristics (ROC) approach, including appraisal of sensitivity, specificity, positive predictive value (PPV), negative predictive value (NPV), and area under the curve (AUC). Optimal score cut-points will be confirmed using the *Youden’s J* statistic (unweighted and weighted prioritize sensitivity). Our target sensitivity and specificity will follow the established standards in screening test research, where test performance is considered acceptable, good, and excellent, at value thresholds of 0.7, 0.8, and 0.9, respectively. In addition, we will use the concordance correlation coefficient (CCC) [55], similar to a weighted κ statistic, to compare the agreement, accuracy, and precision associated with the screening success of the TAPS-ESP. For detecting the CIDI-derived DSM-5 substance use disorder diagnosis, we will examine TAPS-ESP cut-points for the 4-item screener as well as the brief assessment component in detecting levels of problem severity defined on the basis of DSM-5 criteria. As done in the original TAPS study, we will examine cut-points for detecting three different tiers of unhealthy substance use: Problem use (1 + criterion), SUD (2 + criteria), and moderate-to-severe SUD (4–11 criteria). Detection of SUD at any level will be the primary outcome of interest for each substance category. We will also examine performance of the TAPS-ESP in eliciting disclosure of substance use, irrespective of endorsing SUD-related problems.

#### Comparisons by administration format, sex, and age

We will conduct all analyses separately for (a) the interviewer-administered TAPS-ESP, and (b) the self-administered TAPS-ESP. In addition, we exploit the random-order design to directly compare the administration formats in their ability to elicit self-disclosure of substance use, and in their performance with respect to sensitivity, specificity, and ROC curves. We anticipate a fairly balanced representation of male and female participants, as well as adults across the lifespan, and thus will also examine TAPS-ESP performance based on participant sex and age. We will employ a combination of subgroup analyses and multivariable logistic regression models to comprehensively examine these factors. This approach replicates the rigorous analysis strategy of the English language TAPS study [16, 18, 35].

#### Satisfaction and acceptability

Data on satisfaction and acceptability will be examined descriptively, with target benchmarks of ≥ 4/5 for each rating. We will examine whether satisfaction and acceptability ratings differ by participants’ reported substance use behaviors using χ^2^ tests of independence.

### Power

Our power analysis used the approach for minimum sample size determination for sensitivity and specificity analyses as recommended by Bujang and Adnan [56]. These authors computed minimal sample size requirements for screening and diagnostic validation studies under a range of scenarios regarding the prevalence of the disease in the clinical population. Although substance use problems as a whole are prevalent in primary care populations, prevalence is expected to be low for specific SUDs. For a screening study, particularly one with a low prevalence of disease, achieving high sensitivity (i.e., the ability of the test to accurately detect a true case of the condition; true positives) is paramount, and much lower sample sizes are needed for specificity. Thus, we present power calculations for sensitivity as recommended for screening studies, where the prevalence of the disease/condition is low, ranging from 2.5–20%, where power > 0.80 and the null hypothesis is 0.50. Our target sample size of *N* = 1000 exceeds the maximum sample size requirements for screening studies even at a low prevalence of 5%, even if the screening performs only in the “acceptable” range (Table 2). If, as expected, the TAPS-ESP performs in the “good” or “excellent” range (with sensitivities exceeding 0.80 or 0.90), a smaller sample size would be sufficient, even at very low prevalence of 2.5%. The approach detailed above is recommended for screening studies against a diagnostic standard. Nonetheless, we also computed power to detect differences in sensitivity between the TAPS-ESP and other screeners, using the Stata/SE 16 PSS command suite for differences in marginal proportions in dependent samples. We assumed high within-subject correlations and base sensitivity of 0.70 in the comparison screener. At 10% prevalence, power to detect a 10% improvement in sensitivity approached 0.90, but fell below 0.70 when prevalence was set at 5%. Power remained good (0.80) at prevalence of 5% to detect a slightly larger improvement in sensitivity of 15%.

**Table 2:** Minimum sample size for sensitivity in screening studies, with power > 0.80, α = 0.05, and H_0_ = 0.50

Prevalence	H_1_	N_positive_	N_Total_
2.5%	0.70	49	1960
2.5%	0.80	20	800
5%	0.70	49	**980**
5%	0.80	20	400
10%	0.70	49	490
10%	0.80	20	200
20%	0.70	49	245
20%	0.80	20	100

The bolded value of 980 formed the basis for our sample size determination, assuming 5% prevalence and H_1_ = 0.70. N = 1000 was chosen to slightly exceed this target in anticipation of missing data

H_0_ is assumed to be 0.50, as recommended for screening studies. Adapted from: Bujang, MA & Adnan, TH.^56^

### Provider recruitment

Once the TAPS-ESP has completed validation, we will recruit 10 primary care providers to obtain perspectives on barriers, facilitators, and preferences regarding screening with the TAPS-ESP. Participants will be eligible if they are a licensed clinical provider at BSWH (e.g., physician, nurse practitioner, physician’s assistant, clinical social worker), who routinely treats adult patients. Providers will receive training on using the TAPS-ESP resources within the BSWH health information technology system,

### Qualitative interviews

Providers will complete a face-to-face qualitative interview focused on screening and the TAPS-ESP. Interview questions will focus on factors related to providers’ perceived competencies, current screening practices and experiences, and how the TAPS-ESP could fit into the clinical workflow. Interviews will be audio recorded and transcribed for analysis. Data analysis will use a content analysis approach, with themes organized based on the tenets of Kilbourne et al.’s Conceptual Framework for Advancing Health Disparities Research within the Health Care System [46]. Two coders will analyze the data independently and meet to discuss and rectify discrepant interpretations, and ultimately reach consensus on key emerging themes.^82^ In addition, the narratives will be subjected to analysis by a third independent coder using an open coding strategy akin to the first steps in the grounded theory method [57], approaching the dataset without preconceived ideas about what themes and topic areas may be nested within the narrative. The third coder will then meet with the two other coders to discuss the extent of alignment between their interpretations of the data. This approach to triangulation will help to ensure that the qualitative data from providers are examined in a comprehensive way, from multiple vantage points.

## Discussion

This study is an application of a Type 1 effectiveness-implementation hybrid design [58] to a screening validation study, where the effectiveness goal is accurate detection of unhealthy substance use rather than a clinical outcome. The study will yield an approach that could expand access to evidence-based, linguistically accurate, and culturally relevant substance use screening and brief assessment for an underserved health disparity population. To our knowledge, this will be the largest study in the US to validate a Spanish-language screening tool for substance use in primary care. If successful, the TAPS-ESP could have public health impact by enabling evidence-based substance use screening among Spanish-speaking people in the US and could be useful for primary care practices given their increasingly prominent role in addressing substance use problems.

Accurate screening is the first step for delivering health interventions. The US Preventive Services Task Force indicated a critical need for research to establish whether substance use interventions with primary care patients, identified through routine screening, has comparable effectiveness to those seeking treatment and already experiencing medical, social, or legal problems from their substance use [59]. The TAPS-ESP could provide a foundation for developing more effective interventions in primary care among Spanish-speaking populations. This project is timely in its (a) focus on the needs of a growing Spanish-speaking population, (b) acceleration of the diffusion of a new evidence-based screening tool to an underserved group, and (c) application of technology solutions to address disparities in substance use service utilization and outcomes.

### Current study status

Despite some delays in start-up related to late-COVID workflow readjustments and staffing, the validation study was successfully launched at BSWH clinics and recruitment is actively underway. To date, over two thirds of the target sample have been recruited into the study. Based on current staffing and the enrollment flow, we expect to complete recruitment by early 2024. Subsequent to successful validation, future directions for research could include refinement of the TAPS-ESP and implementation research to examine its adoption in real-world clinical practice.

## Data Availability

Not applicable.

## Abbreviations

TAPS: Tobacco, Alcohol, Prescription drug, and illicit Substance use
TAPS-ESP: TAPS Electronic Spanish Platform
SUD: Substance Use Disorder
FQHC: Federally Qualified Health Center
EHR: Electronic health record
DAST: Drug Abuse Screening Test
ASSIST: Alcohol, Smoking and Substance Involvement Screening Test
NIDA-CTN: National Institute on Drug Abuse Clinical Trials Network
CAGE: Cut Down, Annoyed, Guilty, Eye-Opener
AUDIT: Alcohol Use Disorders Identification Test
FTND: Fagerstrom Test for Nicotine Dependence
CIDI: Modified World Mental Health Composite International Diagnostic Interview
ROC: Receiver operating characteristics
PPV: Positive predictive value
NPV: Negative predictive value
AUC: Area under the curve
CCC: Concordance correlation coefficient
